# Atypical dermatomyositis with positive anti-nuclear matrix protein 2 antibodies complicated by rhabdomyolysis syndrome: a case report and literature review

**DOI:** 10.3389/fimmu.2026.1730968

**Published:** 2026-02-04

**Authors:** Zhengyi Zhang, Dawei Jiang, Tao Wei, Kunlan Long

**Affiliations:** 1 School of Clinical Medicine, Chengdu University of Traditional Chinese Medicine, Chengdu, China; 2 Department of Critical Care Medicine, Hospital of Chengdu University of Traditional Chinese Medicine, Chengdu, China

**Keywords:** anti-NXP-2 antibody-positive dermatomyositis, autoimmune disease, case report, literature review, pathological mechanism, rhabdomyolysis syndrome

## Abstract

**Background:**

Idiopathic inflammatory myopathies (IIMs) are a group of systemic disorders characterized by chronic autoimmune dysregulation; their hallmark is symmetric proximal muscle weakness, often with characteristic cutaneous eruptions in dermatomyositis subtypes. Anti–nuclear matrix protein-2 (NXP-2) antibodies are classified as myositis-specific autoantibodies, yet their prevalence remains persistently low. Adult dermatomyositis associated with NXP-2 positivity exhibits a distinctive phenotype in terms of clinical manifestations, complications, and prognosis—particularly an elevated malignancy risk—and is now regarded as an independent subset within the IIMs spectrum. However, reported frequencies vary widely across cohorts, and cases complicated by rhabdomyolysis are exceedingly rare, posing major challenges to timely recognition and optimal management.

**Case presentation:**

We report a 56-year-old man who developed rhabdomyolysis syndrome after a clear precipitating event. Despite aggressive fluid resuscitation, urinary alkalinization with sodium bicarbonate, and continuous renal-replacement therapy to clear myoglobin, systemic manifestations continued to progress. A comprehensive re-evaluation led to the diagnosis of adult anti-NXP-2 antibody-positive dermatomyositis complicated by rhabdomyolysis. High-dose pulsed glucocorticoid therapy combined with supportive measures achieved disease control and gradual stabilization, allowing transfer to a specialized unit in a stable condition. During subsequent follow-up, the patient remained on low-dose glucocorticoids without any significant adverse effects.

**Conclusion:**

Adult anti-NXP-2 antibody-positive dermatomyositis complicated by rhabdomyolysis has been documented only sporadically; its co-occurrence is estimated at <1%, placing it in the ultra-rare category. The precise pathogenic role of this antibody in systemic autoimmunity remains undefined, and systematic evidence linking it to rhabdomyolysis is lacking. Future multi-omic profiling and functional studies are required to determine whether the two disorders share a common mechanistic pathway.

## 1 Introduction

Idiopathic inflammatory myopathies (IIMs), or simply myositis, constitute a rare, heterogeneous family of autoimmune disorders hallmarked by chronic skeletal-muscle inflammation and multi-organ involvement (1). Myositis-specific autoantibodies (MSAs) are central to contemporary classification and prognostication; among them, autoantibodies directed against nuclear matrix protein-2 (NXP-2) define a major subset of juvenile and adult-onset disease (2). Nevertheless, the pathogenic pathways underlying the various MSA subtypes remain incompletely mapped, and their long-term predictive value continues to be refined (3).Population-based surveys estimate an annual incidence of 0.2–2 and a prevalence of 2–25 per 100–000 persons (4, 5), firmly situating IIMs within the rare-disease spectrum. Notably, the frequency and clinical weight of anti-NXP-2 reactivity differ strikingly across ethnic and geographic cohorts (6).

Recent cohort studies have linked NXP-2 positivity to a distinctive phenotype comprising diffuse subcutaneous edema, ectopic calcification, and a severe myopathic syndrome dominated by combined proximal and distal weakness, global myalgia, and marked dysphagia (2, 7). Coexistence of anti-NXP-2-positive atypical myositis and fulminant rhabdomyolysis is exceptionally uncommon; to date, no systematic case report or integrative review has characterized this association. We describe an exceedingly rare instance of IIM with anti-NXP-2 autoantibodies complicated by rhabdomyolysis, with the aim of sharpening clinical awareness and optimizing diagnostic and therapeutic algorithms for this unique presentation.

## 2 Case presentation

A 56-year-old man was admitted to an outside hospital on 20 March 2025 with a 4-day history of sore throat and dysphagia. Physical examination revealed grade-II tonsillar enlargement, dysphagia, and a productive cough with thick white sputum. Laboratory data showed leukocytosis (14.91 × 10^9^/L; reference 3.5–9.5 × 10^9^/L) and neutrophilia (13.59 × 10^9^/L; reference 1.8–6.3 × 10^9^/L). Gram stain of sputum demonstrated abundant Gram-positive cocci (++++, normally absent) and moderate Gram-positive bacilli (++). A diagnosis of acute suppurative laryngitis was made. Despite supportive care, the patient developed progressive odynophagia, trismus, and new-onset generalized pain, prompting transfer to our institution for further management. He had no prior medical history and had been in good health.

On 9 April 2025 the patient was admitted to our ICU with a 20-day history of pharyngeal pain and diffuse myalgia. Physical examination revealed severe pharyngolaryngeal edema, dysphagia, generalized muscle tenderness, oliguria, and cola-colored urine. Laboratory studies showed striking elevations: ALT 246 U/L (ref 9–50), AST 818 U/L (15–40), LDH 1–775 U/L (120–250), CK 11–064 U/L (50–310), serum myoglobin >1–000 ng/mL (3–36), IL-6 33.99 pg/mL (0–4.4) and CRP 39.03 mg/L (0–5). No significant abnormalities were detected in the 15-item male tumor marker panel. Chest CT the same day demonstrated bilateral patchy pulmonary infiltrates. Further history disclosed that the patient had been engaged in heavy manual labor during the 2–3 weeks preceding the onset of initial symptoms (late February 2025), cumulatively lifting loads exceeding 1 tonne. Notably, this period of exertion had ended approximately 3–4 weeks prior to the current presentation. In view of this clear precipitant, rhabdomyolysis complicated by pulmonary infection was diagnosed. Empirical piperacillin–tazobactam was started, together with aggressive intravenous hydration, urinary alkalinization with sodium bicarbonate, and continuous renal-replacement therapy to eliminate circulating myoglobin.

**Figure 1:**
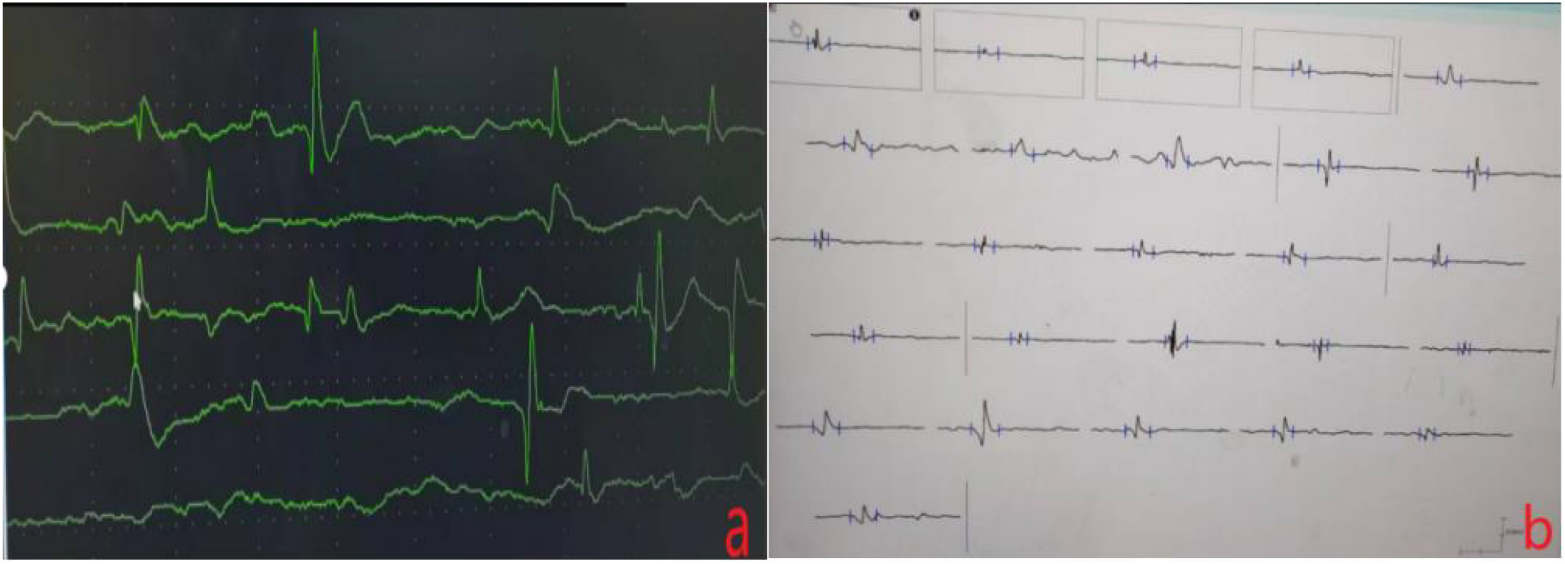
**(a)** Shows fibrillation potentials—abnormal spontaneous activity indicating denervated muscle or unstable sarcolemmal membranes. **(b)** Shows the compound muscle action potential (CMAP): the distal amplitude is normal, whereas proximal stimulation evokes a marked drop in amplitude, indicating conduction block within the muscle.

**Figure 2:**
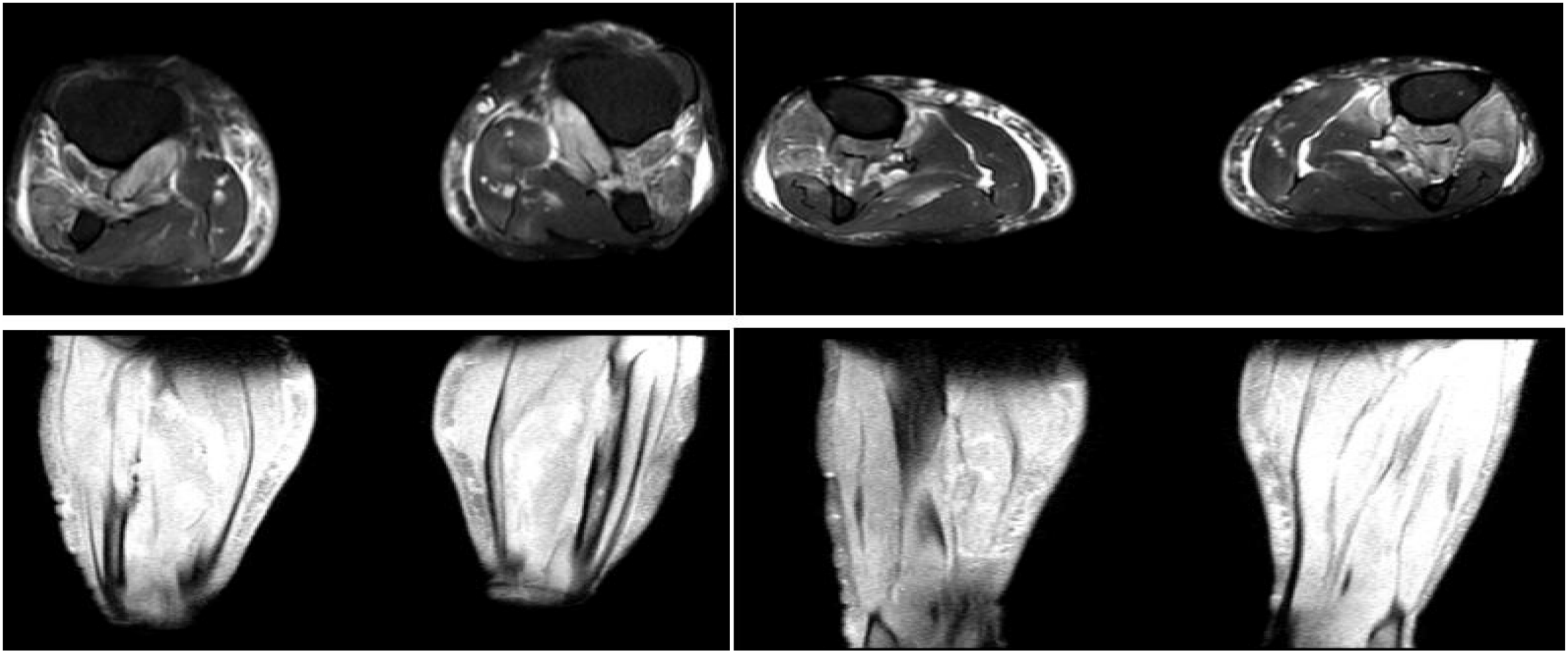
Bilateral lower leg muscles and subcutaneous soft tissues show extensive swelling with patchy slightly long T1 and slightly long T2 signal changes, accompanied by widespread increased and thickened vascular shadows. This non-contrast MRI of both calves strongly suggests active dermatomyositis, particularly the classic imaging triad of combined muscle plus subcutaneous tissue involvement coupled with vascular proliferation.

**Figure 3:**
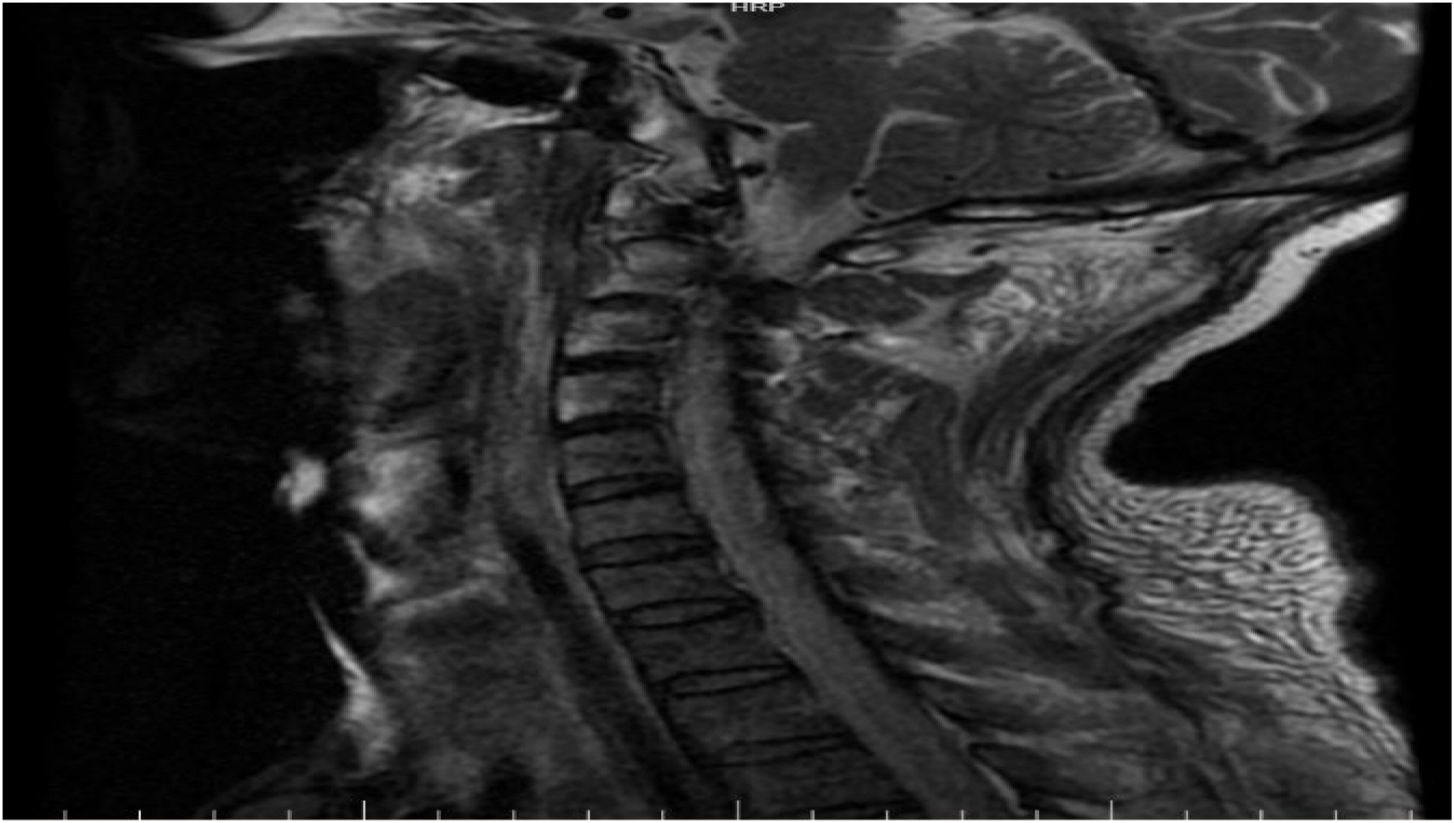
The cervical MRI findings further support active dermatomyositis, with combined posterior cervical muscle and anterior fascial involvement representing a typical imaging pattern of systemic proximal myositis with fascial inflammation. These findings are consistent with the lower-extremity MRI, indicating widespread active disease.

**Figure 4:**
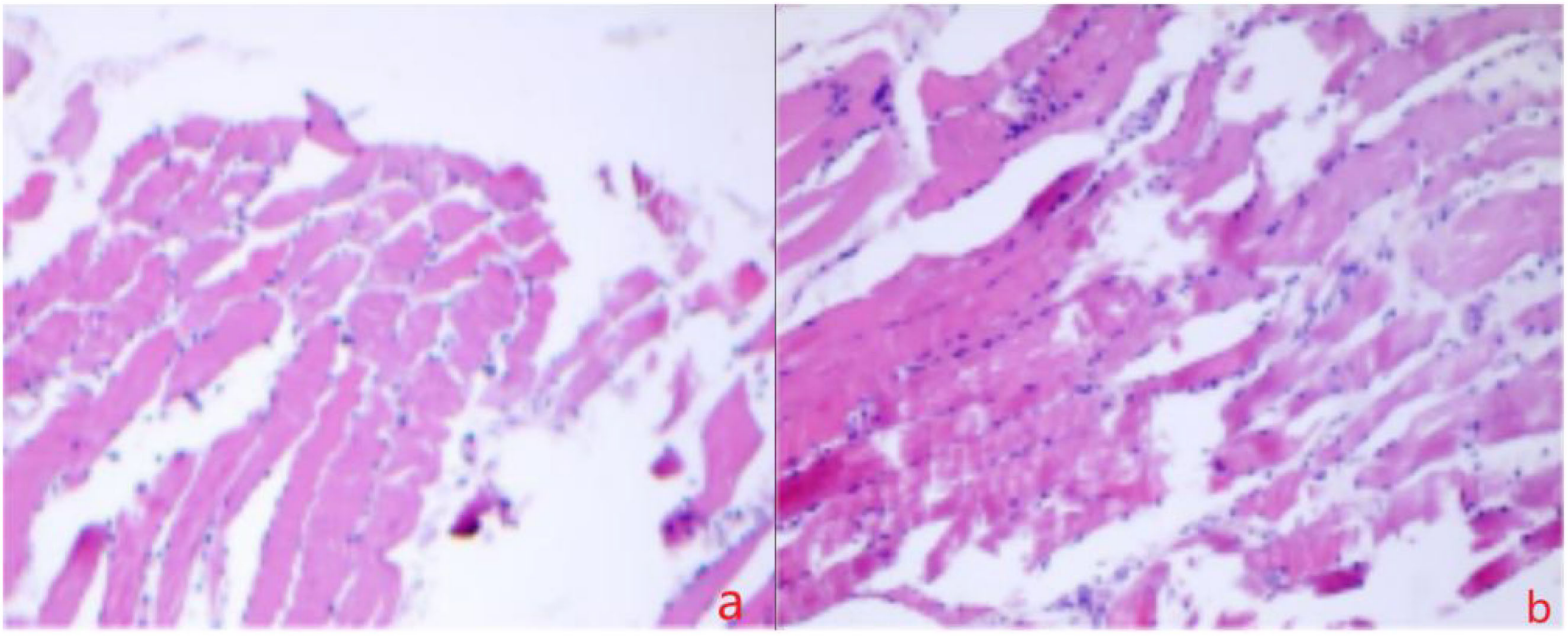
Scattered T lymphocytes (CD3^+^) and plasma cells (CD38^+^) are noted between muscle fibers throughout the entire section; PAS stain is positive. PAS positivity was abolished after diastase digestion, consistent with glycogen accumulation. Congo red staining was negative, ruling out amyloidosis. The presence of scattered inflammatory infiltrates supports an immune-mediated myopathy, with endomysial involvement suggesting autoimmune etiology. PAS positivity indicates possible glycogen accumulation within muscle fibers, consistent with chronic inflammation or metabolic dysfunction. CD38^+^ plasma cells further imply active humoral involvement, reinforcing the rationale for immunomodulatory therapy. These histopathological findings correlate clinically with progressive weakness and elevated creatine kinase levels, underscoring treatment responsiveness in this context.

On 14 April the patient’s generalized pain escalated, accompanied by bilateral upper-extremity edema and rapidly progressive limb weakness that was most pronounced proximally—he could no longer lift his arms above the horizontal. Laboratory values, obtained 6 h after CRRT had been paused, showed a further surge in both inflammatory markers and CK. Repeat chest CT revealed extension of the bilateral pulmonary infiltrates. An emergent multidisciplinary consultation (neurology, rheumatology, and rehabilitation-acupuncture) was convened, and a decision was made to expand the workup and perform an invasive muscle biopsy to clarify the etiology: despite discontinuation of CRRT, creatine kinase (CK) levels surged further, accompanied by rapidly progressive proximal weakness and dysphagia, suggesting ongoing immune-mediated muscle injury rather than simple exertional rhabdomyolysis. Electromyography (14 April) demonstrated a frank myopathic pattern: abundant spontaneous activity in proximal muscles and short-duration, low-amplitude motor-unit potentials on mild contraction (**Figure 1**). Non-contrast MRI of both lower legs showed diffuse intramuscular and subcutaneous edema with patchy hyper-intensity on T1- and T2-weighted sequences, together with prominent, tortuous vascular signal (**Figure 2**). Cervical-spine MRI revealed marked swelling of the posterior cervical musculature and pre-vertebral fascia (**Figure 3**). Myositis-specific antibody profiling (16 April) returned positive for anti-NXP-2 at a titer of 1:100 (**Table 1**). Open biopsy of the left deltoid (17 April) disclosed endomysial and perimysial infiltrates composed of CD3^+^ T cells and CD38^+^ plasma cells, with focal sarcolemmal deposition of PAS-positive material (diastase-sensitive, Congo red negative) (**Figure 4**). These findings established the diagnosis of anti-NXP-2-positive dermatomyositis complicated by rhabdomyolysis. Treatment was escalated to pulse methylprednisolone 80 mg i.v. daily (based on 1 mg·kg^-1^·d^-1^) for 7 consecutive days and intravenous immunoglobulin 20 g daily for 3 days.

**Table 1:** Myositis-specific autoantibody profiling.

Item	Method	Result	Reference range
MSA panel (28-plex)			
Anti-Jo-1 antibody	CBA	Negative (-)	Negative (-)
Anti-PL-7 antibody	CBA	Negative (-)	Negative (-)
Anti-PL-12 antibody	CBA	Negative (-)	Negative (-)
Anti-EJ antibody	CBA	Negative (-)	Negative (-)
Anti-OJ antibody	CBA	Negative (-)	Negative (-)
Anti-Zo antibody	CBA	Negative (-)	Negative (-)
Anti-KS antibody	CBA	Negative (-)	Negative (-)
Anti-Ha antibody	CBA	Negative (-)	Negative (-)
Anti-Mi-2α antibody	CBA	Negative (-)	Negative (-)
Anti-Mi-2β antibody	CBA	Negative (-)	Negative (-)
Anti-TIF1γ antibody	CBA	Negative (-)	Negative (-)
**Anti-NXP2 antibody**	CBA	**Positive (+) 1:100**	**Negative (-)**
Anti-MDA5 antibody	CBA	Negative (-)	Negative (-)
Anti-SAE1 antibody	CBA	Negative (-)	Negative (-)
Anti-SAE2 antibody	CBA	Negative (-)	Negative (-)
Anti-HMGCR antibody	CBA	Negative (-)	Negative (-)
Anti-SRP antibody	CBA	Negative (-)	Negative (-)
Anti-cN1A antibody	CBA	Negative (-)	Negative (-)
Anti-CENP-B antibody	CBA	Negative (-)	Negative (-)
Anti-Scl-70 antibody	CBA	Negative (-)	Negative (-)
Anti-RNA-Pol III antibody	CBA	Negative (-)	Negative (-)
Anti-Th/To antibody	CBA	Negative (-)	Negative (-)
Anti-NOR-90 antibody	CBA	Negative (-)	Negative (-)
Anti-Fibrillarin antibody	CBA	Negative (-)	Negative (-)
Anti-Ku antibody	CBA	Negative (-)	Negative (-)
Anti-PM-Scl100 antibody	CBA	Negative (-)	Negative (-)
Anti-PM-Scl75 antibody	CBA	Negative (-)	Negative (-)
Anti-Ro52 antibody	CBA	Negative (-)	Negative (-)

Bold values in the table indicate positive or abnormal test results.

On 22 April the patient’s sore throat had resolved; swelling of both upper limbs and global muscle weakness had markedly improved, although neck-flexor strength remained reduced. Laboratory tests showed normalization of inflammatory markers, and chest CT demonstrated almost complete resolution of the pulmonary infiltrates. Methylprednisolone was therefore tapered (Reduce by 10 mg per week; when below 20 mg, reduce by 5 mg per week) and methotrexate 10 mg weekly was added. Based on patient preference and in alignment with local integrative practice patterns, the patient received the traditional Chinese medicine formula Bu Zhong Yi Qi Tang as an adjunctive supportive measure, along with daily acupuncture sessions. These interventions were intended to improve general well-being but were not considered evidence-based treatment for dermatomyositis.

## 3 Follow-up

Six months later, the patient reported an excellent quality of life; no cutaneous eruptions, myalgia, dysphagia, or other clinical manifestations had recurred.

## 4 Discussion and literature review

This case illustrates an adult who initially presented with fulminant rhabdomyolysis and was only later recognized to have anti-NXP-2 antibody-positive dermatomyositis. Although standard measures—vigorous hydration, urinary alkalinization and continuous renal-replacement therapy—were instituted promptly, the disease continued to escalate, producing severe muscle inflammation and multi-system involvement. Subsequent myositis-specific antibody profiling, electromyography and muscle biopsy established the diagnosis, leading to high-dose pulsed glucocorticoids combined with intravenous immunoglobulin and an immunomodulator, after which the patient stabilized and improved. This exceptionally rare association underscores that anti-NXP-2-positive dermatomyositis may masquerade as isolated rhabdomyolysis and can trigger a life-threatening muscle necrosis. It also highlights the formidable therapeutic dilemma posed by the simultaneous need for intense immunosuppression and the heightened risk of infection. Early consideration of an autoimmune etiology, rapid initiation of potent immunosuppressive therapy and meticulous infection-control measures are pivotal to reducing mortality in such patients.

Anti-NXP-2 antibody–positive dermatomyositis is a rare autoimmune disorder defined by loss of endomysial capillaries and/or sarcolemmal deposition of the membrane attack complex (8, 9). Clinically it is highly heterogeneous; cardinal features include the characteristic cutaneous signs (heliotrope rash, Gottron papules, Gottron sign), symmetrical proximal limb weakness and dysphagia (10).Delayed diagnosis and treatment frequently result in irreversible muscle damage (11).Paralleling this diagnostic heterogeneity, no standardized therapeutic protocol exists. Current recommendations rest on limited evidence: the cornerstone of idiopathic inflammatory myopathy management remains glucocorticoids combined with conventional immunosuppressants (11–13). Consequently, therapy relies heavily on off-label agents while on-label options are scarce. These regimens can suppress or abolish symptoms, yet they do not target disease-specific pathogenic pathways (11–13) and carry substantial toxicities and therapeutic limitations (14, 15).

Rhabdomyolysis spans a clinical continuum from asymptomatic hyper-CK-emia to life-threatening emergencies characterized by extreme creatine-kinase elevations, electrolyte derangement, acute renal failure and disseminated intravascular coagulation (16). Although our patient had a history of heavy physical exertion, this activity had ended approximately 3–4 weeks before the onset of initial pharyngeal symptoms and 5 weeks prior to the rhabdomyolysis crisis. Crucially, exertional rhabdomyolysis caused purely by muscle trauma typically manifests within hours to days after the precipitating event and resolves rapidly with supportive treatment—a disease course trajectory distinctly different from that observed in our patient. In contrast, this patient’s CK continued to climb after cessation of CRRT, accompanied by proximal weakness, dysphagia, myopathic EMG changes and muscle-biopsy evidence of CD3^+^/CD38^+^ inflammatory infiltrates—findings that point to ongoing immune-mediated myofiber injury. Therefore, we speculate that the temporally remote lifting event may have initiated myofiber damage through subclinical inflammation, but given the 3–4 week interval, it was neither a triggering nor a determining factor. The persistently elevated CK levels despite discontinuation of CRRT, accompanied by proximal muscle weakness, dysphagia, myopathic EMG changes, and evidence of CD3^+^/CD38^+^ inflammatory infiltrates on muscle biopsy, all suggest that ongoing immune-mediated myofiber injury was the primary driver. This interpretation aligns with the handful of previously reported IIM cases complicated by rhabdomyolysis in which immune dysregulation alone, without overt external stressors, precipitated acute myofiber disintegration (17, 18). Consequently, non-traumatic rhabdomyolysis should always prompt clinicians to include inflammatory myopathy in the differential diagnosis and to proceed promptly with appropriate auto-immune serology (19).In anti-NXP-2 antibody–positive dermatomyositis, NXP-2—a scaffold protein tethered to the nuclear matrix and intimately associated with the nuclear-pore complex—is recognized by autoantibodies (20).Complement activation then drives formation of the membrane-attack complex (MAC), which inserts directly into the nuclear envelope, triggering Ca²^+^influx and calpain activation. Calpain cleaves nuclear lamins and nesprin-1, disrupting envelope integrity; released HMGB1 amplifies inflammation via the TLR4/NF-κB loop, while Ca²^+^ overload opens the mitochondrial permeability-transition pore, releasing cytochrome c and activating the caspase-9/3 apoptotic pathway. Persistently active calpain further degrades titin and dystrophin, leaving sarcomeres in a “sub-necrotic” state (21). When a second hit such as strenuous exercise exhausts ATP, Ca²^+^pumps fail, Ca²^+^homeostasis collapses, and extensive fiber breakdown ensues—clinically manifesting as classic rhabdomyolysis. This sequence has been directly validated in recent NXP-2–/– mice and passive-transfer models, which show nuclear-envelope rupture, MAC deposition, and myocyte necrosis (22), indicating that anti-NXP-2 antibodies are not merely diagnostic but pathogenic drivers of rhabdomyolysis by destabilizing the nuclear envelope and amplifying the mitochondrial–Ca²^+^overload cycle. Nuclear-envelope damage also impairs DNA repair and genomic stability, further compromising stress recovery and predisposing fibers to irreversible injury under metabolic stress. This pathogenic cascade explains why some dermatomyositis patients develop acute myolysis without prominent inflammation, and provides a rationale for early therapeutic blockade of TLR4 signaling or modulation of Ca²^+^homeostasis to halt or delay muscle structural collapse.

## 5 Research hotspots and frontiers

### 5.1 Mitochondrial function

Mitochondrial dysfunction—including mtDNA depletion and impaired bioenergetics—is a central feature of the idiopathic inflammatory myopathies (IIMs) (23, 24), positioning mitochondria as a potential therapeutic target. Mitochondria isolated from human umbilical-cord mesenchymal stem cells (PN-101) have shown promising efficacy. Kim JY et al. demonstrated that PN-101 not only promotes mitochondrial repair and myogenic differentiation, but also suppresses immune-cell infiltration and pro-inflammatory cytokine expression in C-protein-induced murine myositis, while restoring metabolic homeostasis (25). A subsequent prospective clinical trial further revealed that PN-101 is safe and well tolerated; adults with refractory polymyositis or dermatomyositis achieved at least minimal improvement in total improvement score (TIS) from baseline (25).Adenosine-receptor signaling—especially via the A2B subtype—regulates lymphocyte differentiation, activation and immune homeostasis (26). Zhou Y and Gnad T et al. reported that A2B agonism attenuates skeletal-muscle injury and inflammation *in vivo* and improves muscle strength (27, 28). Mechanistically, Zhou Y et al. clarified that A2B activation down-regulates HIF-1α, shifts the Th17/Treg balance and prevents Treg exhaustion, thereby markedly ameliorating experimental autoimmune myositis (27).Immunomodulation and metabolic remodeling converge mechanistically in IIM pathology. Strategies that bolster mitochondrial function—whether by mitochondrial transplantation or receptor-mediated enhancement—may simultaneously suppress muscle inflammation and restore mitochondrial integrity while promoting regenerative myogenesis, offering a novel dual-pronged therapeutic approach for patients with IIMs.

### 5.2 B-cell targeted therapy

B-cell–targeted intervention is emerging as a highly promising therapeutic strategy for the idiopathic inflammatory myopathies (IIMs) (29). Transcriptomic profiling reveals that B cells from IIM patients exhibit pronounced endoplasmic-reticulum stress and enrichment of oxidative-phosphorylation pathways, indicating a hyper-activated phenotype. These activated B cells sustain a positive-feedback loop by secreting cytokines (e.g., IL-6, IFN-α) and immune-regulatory factors that reciprocally activate dendritic cells and T cells, thereby perpetuating chronic immune-mediated muscle injury (30, 31). Selective depletion or functional silencing of B cells therefore breaks this loop and ameliorates both skeletal-muscle and systemic damage.

CD19 is a lineage-restricted surface antigen expressed from pro-B cells through mature B cells. After a single infusion of CD19-targeted CAR-T cells, IIM patients achieve rapid peripheral B-cell aplasia, accompanied by improved muscle strength, falling autoantibody titers and concomitant glucocorticoid taper, demonstrating rapid and durable disease control (32, 33). Analogous results have been observed in systemic lupus erythematosus—an autoimmune disorder that overlaps with the IIM spectrum—where CD19 CAR-T monotherapy induced drug-free remission in refractory cases (34), providing direct proof-of-concept for deep B-cell depletion in IIMs. For more mature CD20^+^ B-cell subsets, rituximab has consistently reduced disease activity in randomized and real-world studies, with particular efficacy in antisynthetase-antibody-positive and dermatomyositis subtypes (35).

Beyond direct B-cell depletion, blockade of survival and differentiation factors offers an indirect yet precise means of suppressing pathogenic B-cell responses. B-cell activating factor (BAFF) and a proliferation-inducing ligand (APRIL) are critical for B-cell homeostasis; serum and muscle-tissue levels of BAFF/APRIL are significantly elevated in IIM patients and correlate positively with autoantibody titers and global disease activity. Inhibitors targeting the BAFF/APRIL axis—e.g., belimumab (anti-BAFF) and atacicept (BAFF/APRIL dual trap)—can therefore be used as monotherapy or in combination to fill the “blank zone” between profound depletion and fine functional modulation, enabling a precision continuum from “deep clear” to “rheostat” control of pathogenic B-cell responses (36).

### 5.3 Non-coding RNA

Non-coding RNAs (ncRNAs) orchestrate key processes in idiopathic inflammatory myopathies (IIMs), including the propagation of inflammation, skeletal-muscle regeneration and differentiation, the titers of disease-specific autoantibodies, and even myalgia perception. As readily detectable biomarkers, ncRNAs can both reflect and modulate the course of immune-mediated muscle disease (37, 38). MicroRNAs operate through a dual rheostat that determines myofiber fate. On the one hand, pro-inflammatory miR-155 and miR-21 are rapidly induced by TNF-α/IL-1β; they directly target myogenic regulatory factors such as MyoD and MEF2C, thereby arresting myoblast-to-myotube fusion. On the other hand, counter-regulatory miR-146a and miR-26a dampen NF-κB and p38 MAPK signaling, mitigating inflammation and rescuing the differentiation program (39). Consequently, accurate identification and selective modulation of disease-relevant ncRNAs have emerged as a therapeutic imperative in IIMs. Yang Y et al. recently summarized evidence that ncRNAs also mediate the beneficial effects of exercise in IIM patients—most notably by accelerating post-inflammatory muscle regeneration and alleviating disease-associated pain (11). Unfortunately, current insights remain largely descriptive and tissue-based; future work must elucidate the detailed molecular circuitry governed by ncRNAs and translate these findings into rationally designed ncRNA-targeting drugs.

## 6 Limitations

The patient first sought medical care for sore throat and dysphagia. Because classic cutaneous lesions were absent, an autoimmune etiology was not entertained initially; this diagnostic gap delayed appropriate immunosuppression and allowed disease progression. The present case has several limitations:①Although the 3–4 week interval between exertion and symptom onset makes pure exertional rhabdomyolysis unlikely, we cannot be completely certain that prior physical activity did not exert a priming effect on the muscle. ②We observed only a temporal association between anti-NXP-2 positivity and rhabdomyolysis; functional or mechanistic studies *in vitro* or in animal models are lacking, and a direct causal link has not been established. ③Follow-up was limited to three months; long-term relapse rate, chronic organ outcomes and potential adverse effects of prolonged immunosuppression were not assessed, restricting a comprehensive understanding of the natural history and prognosis of anti-NXP-2-positive dermatomyositis. ④During the convalescent phase, the patient received Bu Zhong Yi Qi Tang and acupuncture based on personal preference and institutional integrative care protocols. It is important to emphasize that these interventions lack established efficacy in anti-NXP-2-positive dermatomyositis or rhabdomyolysis, and their mechanisms of action have not been validated through rigorous immunologic studies. We do not attribute the patient’s clinical improvement to these therapies; rather, they are reported to provide complete transparency regarding the care delivered. Future investigations would require randomized, controlled trial designs to evaluate any potential role in symptomatic support or quality-of-life outcomes.

## 7 Conclusion

As a single-center case report, this study is based solely on the clinical data of one 56-year-old male. Individual baseline characteristics, comorbidities and treatment response may deviate markedly from the broader population, so the findings have limited generalizability. The presence of an obvious precipitant—heavy muscular overexertion—must not dissuade clinicians from systematically screening for idiopathic inflammatory myopathy; anti-NXP-2 antibody-positive dermatomyositis can begin insidiously and evolve rapidly into life-threatening rhabdomyolysis. Early recognition of an autoimmune origin, prompt initiation of intensive immunosuppression and meticulous infection control are pivotal to reversing disease course and reducing mortality. Adjunctive therapies without established disease-modifying effects should be clearly distinguished from evidence-based immunosuppressive treatment. For such patients, even when initially presenting with rhabdomyolysis, comprehensive malignancy screening and long-term surveillance should be initiated. Multi-center, prospective cohorts that include patients with diverse etiologies, disease stages and treatment backgrounds, together with long-term follow-up of multi-organ outcomes, are needed to establish a causal link between the anti-NXP-2 subtype and rhabdomyolysis/systemic damage, and to develop an evidence-based, precision treatment pathway and life-long management strategy.

## Data Availability

The original contributions presented in the study are included in the article/Supplementary Material. Further inquiries can be directed to the corresponding author.
